# Symptoms reported by Canadians posted in Havana are linked with reduced white matter fibre density

**DOI:** 10.1093/braincomms/fcac053

**Published:** 2022-03-07

**Authors:** Guillermo Aristi, Lyna Kamintsky, Margaux Ross, Chris Bowen, Cynthia Calkin, Alon Friedman, Javeria A. Hashmi

**Affiliations:** 1 Department of Anesthesia, Pain Management & Perioperative Medicine, Dalhousie University, Nova Scotia Health Authority, Halifax, Canada B3H 1V7; 2 Departments of Medical Neuroscience, Pediatric and Surgery, Dalhousie University, Nova Scotia Health Authority, Halifax, Canada B3H 4R2; 3 Department of Psychiatry, Dalhousie University, NSHA, Halifax, Canada B3H 1V7; 4 Department of Diagnostic Radiology, Dalhousie University, NSHA, Halifax Canada, B3H 1V7; 5 Department of Brain and Cognitive Sciences, Zlotowski Center for Neuroscience, Ben-Gurion University of the Negev, Beer-Sheva 1084105, Israel

**Keywords:** brain injury, diffusion-weighted MRI, Havana syndrome, headache, white matter

## Abstract

Diplomats representing the USA have reported with unusual neurologic symptoms and MRI changes after being posted in Havana, Cuba between late 2016 and 2018. Here, we examined white matter microstructure and network connectivity of individuals stationed in Havana, using diffusion-weighted MRI, fixel-based analysis and structural connectomics as implemented in MRtrix3. MRI data acquisition and clinical assessments were done in a total of 24 diplomats and their family members and 40 healthy controls. The diplomat data were grouped into an exposed cohort (*n* = 16) and an unexposed cohort (*n* = 10), and among these, two individuals were assessed before and after potential exposure. Fixel-based analysis revealed a reduction in fibre density in two specific regions: the fornix and the splenium, in exposed individuals, relative to unexposed individuals and healthy controls. *Post hoc* analyses showed the effect remained present (*P* < 0.05) in both regions when comparing exposed and unexposed diplomats; and reduced fibre density was correlated with longer time period stationed in Cuba after age correction. Reduction of fibre density was also found to be linked with clinical symptoms of persistent migraine, tinnitus, sound sensitivity and fatigue. Network statistical comparisons revealed decreased structural connectivity in two distinct networks, comprising subcortical and cortical systems in exposed individuals, relative to unexposed and normative data. While the cause for the differences between the groups remains unknown, our results reveal region-specific white matter injury, that is, significantly correlated with clinical symptoms.

## Teaser

Aristi *et al*. report that the neurologic symptoms reported by Canadian individuals who were posted in Havana between 2016 and 2018 are significantly associated with lower density in white matter pathways. The specific cause for the observed white matter differences remains unknown.

## Graphical Abstract

**Graphical Abstract:**
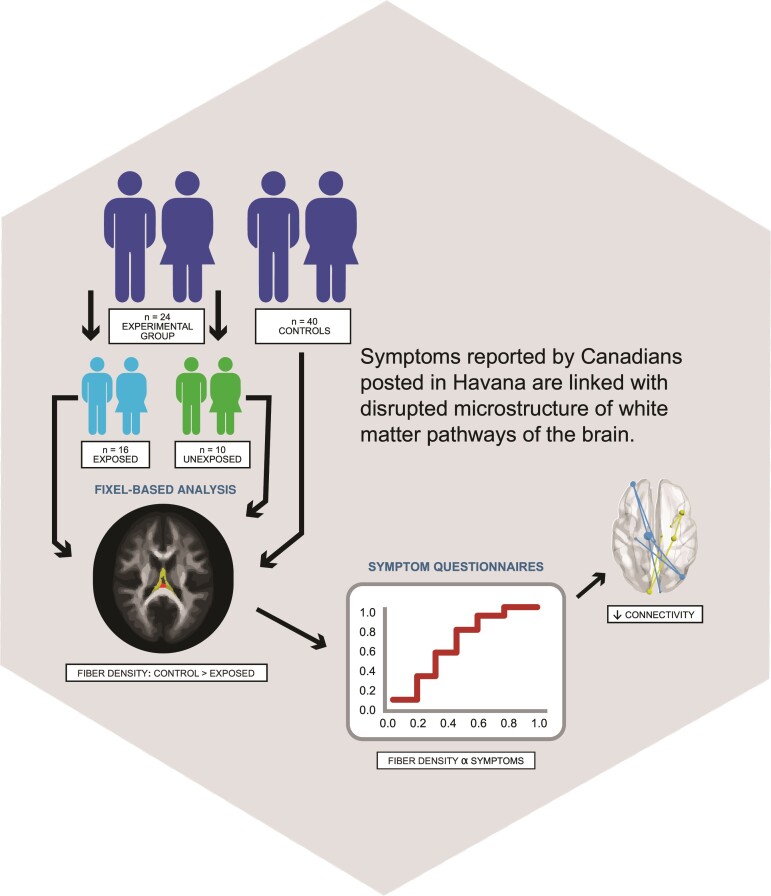

## Introduction

In 2016, the US diplomats in Havana began reporting unusual sensory and auditory stimuli accompanied by symptoms of dizziness, tinnitus and various cognitive manifestations.^1^ A follow-up ∼6–7 months later revealed lasting abnormalities in cognitive, vestibular and oculomotor domains.^2^ The variety of presenting symptoms proved difficult to diagnose and have prompted many to speculate regarding the source of injury.^3^ A similar set of symptoms was later reported by the Canadian diplomats stationed in Havana and their families.^4^ A multimodal assessment of clinical and psychological symptoms revealed that the symptoms were similar to those of the American diplomats. The cause for these changes remains unclear and this ambiguity necessitates an investigation into the relationship between neural effects and the symptoms.

Neuroimaging abnormalities associated with the presence in Havana were identified in both US and Canadian participants. US studies have identified differences in grey and white matter (WM) volume, cerebellar diffusion properties and functional network connectivity.^5^ In a cohort of 26 Canadian diplomats and their family members, our group has previously reported the evidence of abnormalities in WM (examined using diffusion-weighted MRI, dMRI), blood-brain barrier integrity and brain electrical activity (studied using magnetoencephalography).^4^ The studies of US diplomats have highlighted the resemblance of the observed symptoms to the sequela of traumatic brain injury (TBI).^2,5^ However, to date there is no consensus regarding the cause of the symptoms, and the proposed aetiologies include both those of internal and external origin.^3,4^ Hence, the identification of the potential neurological origins of the symptomatology may help the treatment of patients and prevention of future injuries.

In the present study, we focus on WM abnormalities in the exposed Canadians, examining both (i) focal microstructural integrity, and (ii) whole-brain network structural connectivity. Microstructural WM changes are indicative of axonal injury, and considered to be a hallmark of brain damage.^6^ While such microstructural abnormalities are not visible in routine clinical MRI, dMRI allows the delineation of WM fibres in the brain. Diffusion-weighted data can be acquired and analysed using several paradigms, with diffusion tensor imaging (DTI) and tract-based spatial statistics (TBSS) being the most commonly used approach. This method has been shown to detect subtle WM changes related to injuries, including TBI,^7,8^ and was previously used to demonstrate that certain WM regions were affected in a cohort of American diplomats.^5^ However, emerging evidence highlights the limitation of this technique, especially its unreliability in analysing voxels with crossing fibres, which represent more than 70% of WM.^9^ To overcome this limitation, here we used a method combining high angular resolution^10^ with the recently developed statistical analysis method called fixel-based analysis (FBA). This technique uses constrained spherical deconvolution (CSD) to provide micro- and macro-structure estimations for individual WM tracts in a voxel, modelled as ‘fixels', which refers to a specific fibre population within a single voxel.^11,12^ FBA uses the metrics fibre density (FD) and fibre cross-section (FC) to capture microstructural differences pertaining to axon density, and macrostructural differences pertaining to fibre morphometry, respectively. Fibre density and cross-section (FDC) is used to capture a combination of the two properties.^12^ This technique has now been demonstrated to have the required sensitivity to detect changes with clinical diffusion-weighted acquisitions.^13,14^ To verify the observed effects and establish the sensitivity of this method, we also analysed the data using TBSS and test for reproducibility of the TBSS-based findings reported from the American cohort.^5^

Since axonal injury is often diffuse, affecting widespread structural pathways throughout the brain,^15^ we also examined alterations in whole-brain network architecture, using probabilistic tractography and network-based statistics (NBS).^16^ Thus, in addition to FBA, we tested whether whole-brain network architecture of WM pathways is affected in the Canadian diplomats and their families.

The goal of the present study is to provide a detailed characterization of WM changes in Canadian diplomats deployed in Havana during 2016–18, and to examine the correlation between WM abnormalities, participants’ symptoms and time of posting in Havana. Several techniques were employed for quantifying WM abnormalities, in order to cross-validate evidence of WM injury. The used approaches examined: (i) focal microstructural WM abnormalities, using FBA and corroboration with fractional anisotropy (FA) and TBSS; and (ii) changes in whole-brain network architecture. All associations were tested with statistical rigour, including corrections for multiple comparisons and age;^17^ and several different WM analysis techniques.

## Materials and methods

### Participants

The study was approved by the Dalhousie University Research Ethics Board, and all participants provided consent in accordance with the Declaration of Helsinki. A total of *n* = 24 (14 female; mean ± standard deviation age 43.83 ± 11.04 years) participants volunteered to participate in the study through referral from Global Affairs Canada. Adult Canadian’s were referred by the Global Affairs Canada for evaluation between the period of 10 August 2018 and 20 February 2019. All individuals agreed to participate and were categorized as exposed or non-exposed based on whether they had been on ground in Havana. Exposure of a participant was determined if, at the time of assessment, the participant had spent a time period of at least 30 days in Cuba within the period of 2016–19, when neurological symptoms of unknown origin were the first reported.^1^ In this work, ‘exposure' strictly refers to exposure to Havana.

The exposed cohort consisted of *n* = 16 [9 females; age 39.81 ± 10.03 years and the unexposed cohort consisted of *n* = 10 (6 females; age 47.4 ± 12.11 years)]. Among these groups, three individuals who were assessed before and after exposure, therefore, for these subjects, only the data collected after exposure were used for the diffusion analysis leaving us with an *n* = 8 in the unexposed cohort. A separate group of healthy participants (unexposed, non-referred/non-diplomats *n* = 40) were added to the control group to build a more robust statistical model so that we could properly test for abnormalities related to exposure. This non-referred unexposed group included *n* = 40 normative healthy participants (22 female; age 31.9 ± 10.03 years) from another study.^18^

### Inclusion/exclusion criteria

For details on participants and different types of testing (see Friedman *et al.*^4^ for details) Participants were excluded from the study if (i) MRI was contraindicated (subject had metal implant, pacemaker, biostimulator, neurostimulator, internal defibrillator, history of metal in eye, inner ear implant, cerebral aneurism clip, joint replacement, any known metal in their body or were pregnant or breastfeeding); or (ii) subject lacked full capacity to consent to study participation as determined by study clinicians. All referred participants provided their consent. On page 8, we state that none of the screened participants met the exclusion criteria for the DWI aspects of the study. Additional details on clinical metrics such as blood test results are available in a freely available preceding report.^4^

### Comorbidities

Comorbidities and pre-existing conditions related to participant neurological history and previous psychiatric diagnosis include the following: None of the healthy controls (non-referred participants) had a history of pain, head injury or neuropsychological disorders. Among the exposed individuals, there were reports of previous headaches, which were frequent in one participant, occasional headaches in one participant and migraines in two participants. None of the other participants had a history of headaches. In addition, five participants reported past history of head injury, two participants reported prior reports of loss of consciousness, in one participant there was a history of seizures and in one participant, there was report of seasonal affective disorder. Among non-exposed control participants, there were no reports of headaches or migraines. However, in the non-exposed individuals, three participants that had a past history of head injury, and two participants had a history of loss of consciousness in. One participant who was tested before and after exposure reported the previous history of frequent headaches.

### Symptom assessment

A profile of symptoms was collected for all diplomats and their families as part of the recruitment’s initial screening. The presence of neurological symptoms was determined by medical and a battery of self-rated questionnaires. The questionnaires used included the Beck Depression Inventory-II and Beck Anxiety Inventory,^19,20^ Modified Mini Screen^21^ Headache Impact Test,^22^ Migraine Disability Assessment Test,^23^ Post-Traumatic Stress Disorder Checklist—Civilian,^24^ Pittsburgh Sleep Quality Index^25^ and the Rivermead Post-Concussion Symptoms Questionnaire.^26,27^

Supplementary Table 1 includes information on the duration of exposure and time between exposure and assessment. The assessment completion date signifies that all testing was completed by that date. MRI images and assessments were completed within a period of 2 days.

### MRI data acquisition

MRI images acquired for all participants included diffusion-weighted images and structural T_1_-weighted images. dMRI images were acquired using a 32-channel head coil on a 3-T GE MR750 MRI scanner with the following specifications: oblique-axial single-shot echo-planar imaging sequence, *b* = 1000 s/mm^2^, 60 directions, field of view (FOV) = 22 cm, 77 axial slices, isotropic voxel resolution of 2 × 2 × 2 mm^3^, TR = 8 s and acceleration factor of 2. Interleaved *b* = 0 s/mm^2^ images were acquired to facilitate motion correction and an additional scan of seven *b* = 0 s/mm^2^ reverse-phase encoded polarity images were acquired to facilitate image distortion correction. T_1_-weighted images were acquired with the following specifications: TR = 4.4 ms, TE = 1.908 ms, FOV = 224 × 224 mm, voxel size = 1 mm^3^, flip angle = 9°.

### MRI image preprocessing

Preprocessing pipelines for diffusion data were carried out using the MRtrix3 software.^28^ Preprocessing included denoising; removal of Gibbs ringing artefacts; and correction of off-resonance fields induced distortions, eddy currents and head motion using the FSL toolbox.^29^ T_1_-weighted structural images were registered to subject’s corresponding mean *b* = 0 s/mm^2^ diffusion-weighted image, followed by co-registration to standard space using ANTS software^30^ to warp a parcellation scheme from Montreal Neurological Institute standard space to subject diffusion space. The parcellation scheme used was an edited version of the Harvard–Oxford parcellation^31^ consisting of 131 regions. Finally, structural images were segmented into grey matter (GM), sub-GM, WM and CSF using FSL for implementing the anatomically constrained tractography (ACT) framework.^32^

### Fixel-based analysis

Bias field correction and global intensity normalization were done across the normative healthy participants using group-wise registration, followed by CSD to obtain single-fibre WM response functions. The mean response function was used to compute fibre orientation distribution (FOD) images and to create a study-specific FOD template using a voxel size of 1.3 × 1.3 × 1.3 mm^3^. Diplomats’ data were incorporated to the cohort by applying bias field correction and normalizing to the normative healthy participants before computing FOD images.

Following registration of participants’ FOD images to the template, subject masks were warped to obtain an intersection template mask, which was then used to generate the fixel template mask. The FOD lobes were thresholded at 0.30 to constrain fixels to most WM fibre populations in the brain. Next, FD values were obtained for all participants and mapped to template fixels, followed by computation of FC using subject-to-template matrix warps, and FDC by multiplying the two metrics. A tractogram of the FOD template was done using probabilistic tractography with an FOD amplitude cutoff of 0.1 to generate 20 million streamlines, followed by SIFT^33^ filtering to 2 million streamlines

Group statistical comparisons were performed independently on FD, FC and FDC for every fixel using a generalized linear model to investigate microstructural differences in WM tracts between the unexposed cohort (unexposed diplomats and families plus healthy controls) and the exposed cohort (exposed diplomats and families). Image data acquired before the exposure of two individuals were excluded from the design to avoid a group comparison with partially paired data set. Statistical analysis was conducted using connectivity-based fixel enhancement, non-parametric permutation testing using 5000 permutations and family-wise error correction (FWE).

After running a whole-brain comparison between all healthy unexposed participants against the exposed participants, we directly compared the referred unexposed diplomats with the exposed diplomats as a *post-hoc* comparison. The fibre density values from the unexposed control participants were separated into their respective groups (referred versus non. referred), and compared with the exposed group.

### Further analysis of fornix and splenium disruption

Fixels that mapped as significant (*P* < 0.05) from the FBA, as reported in Friedman *et al.*,^4^ were identified as corresponding to the posterior region of the fornix and the splenium of the corpus callosum. The significant fixel map was hence used to generate two masks for the two regions of interest (ROIs) to obtain mean FD values for the two affected regions separately for further analysis.

Disruption of FD was assessed using unpaired *t*-tests in both splenium and fornix between exposed and non-exposed diplomats with the exclusion of pre-exposed data sets, and we investigated the relationship between FD disruption and the time period individuals were stationed in Havana since fall 2016 while controlling for age using partial correlations.

Receiver operating characteristic (ROC) analyses and Student’s *t*-test were used to assess the link between microstructural differences in FD of diplomats and clinical symptoms. We did this to discern how the WM tract disruption could be linked to a symptom (e.g. headaches) and if participants with a specific symptom had distinctly decreased fibre microstructure as compared with those did not present that symptom.

### Structural connectivity assessment with ACT and SIFT2

ACT was done for all images using whole-brain probabilistic tractography to seed a total of 10 million streamlines per participant. This tractography method used outputs from the tissue segmentation of structural images, as implemented in FSL, to seed randomly in WM; use of the iFOD2 algorithm with an FOD cutoff of 0.1 and streamline backtracking. Given analysis results reported by Friedman *et al.*^4^ included disruption of the fornix, a fornix mask in standard space was warped and merged to the WM segmentation of all individuals in diffusion space prior to seeding streamlines using ACT because of frequent mis-segmentation of the fornix into CSF or GM using FSL’s FAST and FIRST. Subsequently, spherical deconvolution filtering of tractograms (revised) (SIFT2) was used to reduce the biases in the reconstructed data by weighting each streamline with a cross-sectional area multiplier as determined by the individual streamline’s contribution to the estimated diffusion signal.^34^

### Connectome construction

Structural connectomes were created using resulting SIFT2-weighted streamlines to estimate edge weights and an edited version of the Harvard–Oxford atlas consisting of 131 ROIs^31^ to delineate brain regions. Streamline contribution to network edges was defined as the number of streamlines connecting end-to-end two regions that were less than 2 mm from the streamline end-point.

### Data availability

The data that support the findings of this study are available on request from the corresponding author. The data are not publicly available due to the high specificity of the inclusion criteria in the exposed cohort.

## Results

### Fornix- and splenium-specific changes in FD are directly linked to posting in Havana

**Figure 1:**
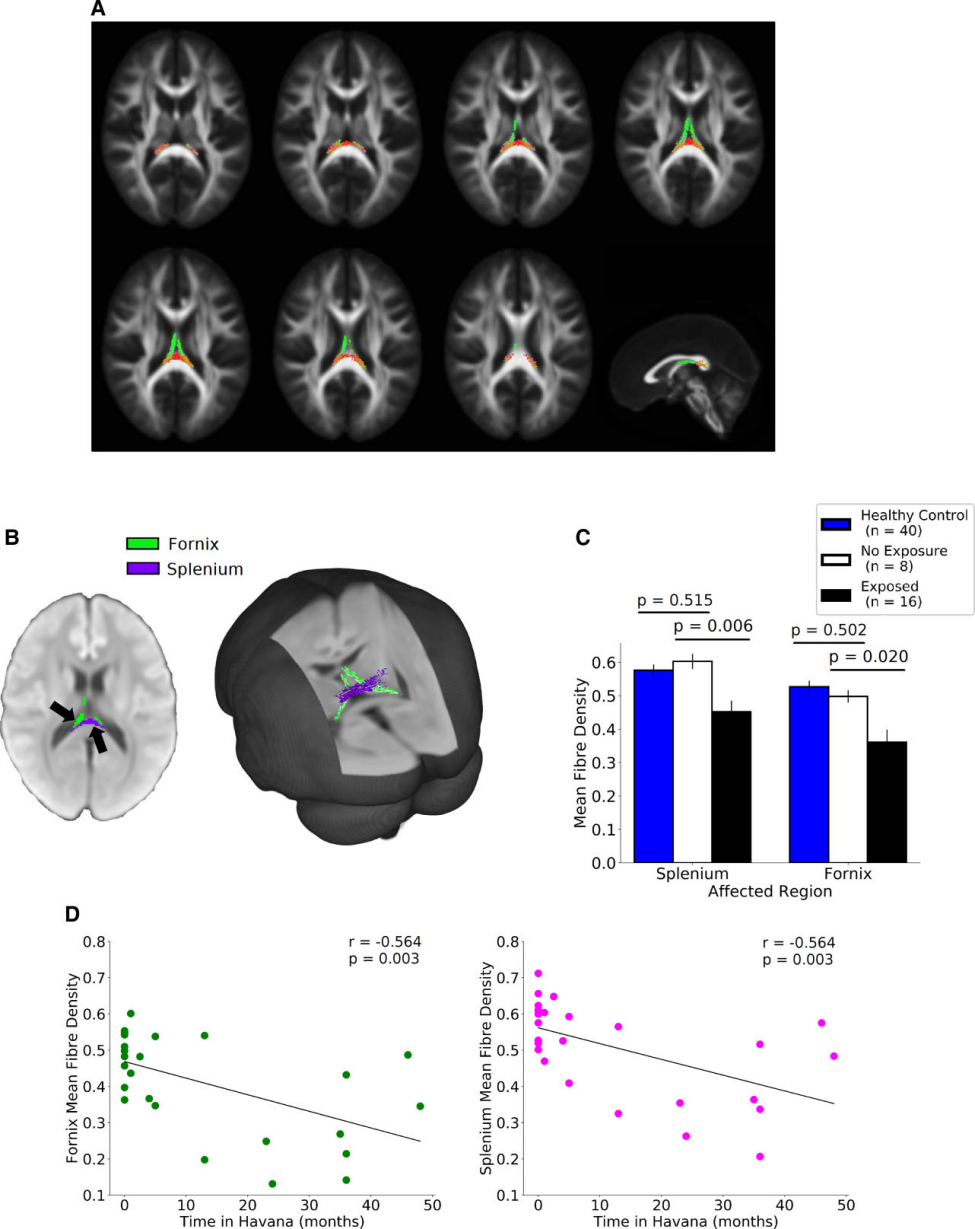
**Fixel-based analysis reveals region-specific changes to the fornix and splenium in Canadian diplomats and their families stationed in Havana, Cuba.** (**A**) Streamlines coloured by direction show the white matter regions affected in the cohort exposed in Havana.^4^ (**B**) A decrease in fibre density was observed predominantly along the right crus of the fornix, past the hippocampal commissure and projecting into the hippocampus, as well as in the splenium of the corpus callosum. (**C**) *Post hoc* analysis showed a significant (*P* < 0.05) decrease in FD in both white matter regions (fornix and splenium) between unexposed healthy controls, unexposed referred group and the exposed group. (**D**) Analysis revealed a significant, age-correctedcorrelationbetween FD of fornix and splenium with duration of posting in Havana since fall 2016.

Preliminary findings from a whole-brain group comparison using FBA were reported by Friedman *et al.*,^4^ and revealed that FD was significantly decreased in a region comprising the fornix and splenium (Fig. 1A and B).

Here, we examined the patterns of WM injury in the splenium and fornix to determine whether the observed reduction in FD was correlated with the duration of posting in Havana. We found that compared with individuals who were not stationed in Havana, those who did spend more than 30 days there since fall 2016 had significantly lower FD in both the splenium and the fornix (Fig. 1C). Participants in the three groups (unexposed healthy controls, unexposed referred group and exposed groups) showed significant differences in fibre density. There were no significant differences between the two unexposed groups (*P* > 0.05). The *post hoc* differences between unexposed referred and exposed groups were significant for both the splenium and the fornix (Fig. 1C). Moreover, the observed loss of FD was significantly correlated with the number of months spent in Havana (Fig. 1D): the more time individuals had spent in Havana, the lower FD they had in both the splenium (*r* = −0.564, *P* = 0.003) and the fornix (*r* = −0.564, *P* = 0.003).

While an analogous analysis with FA and TBSS revealed no significant difference in WM tracts between the groups (family-wise error correction, FWE *P* = 0.16), voxel clusters with the highest significance (0.16 < FWE *P* < 0.30), were observed in the splenium of the corpus callosum, the fornix and the left superior corona radiata (Supplementary Fig. 1 and Table 2). A previous investigation on American diplomats has reported significant changes in the cerebellar WM by using DTI and correction for multiple comparisons localized to the cerebellum.^5^ The study showed the American cohort to have significantly decreased mean diffusivity (MD) in the inferior vermis of the cerebellum.^5^ To test the reproducibility of this finding in the Canadian population, we used DTI and measured MD in the cerebellum using a correction procedure circumscribed to the cerebellum. The MD analysis of six functionally defined regions in the cerebellum showed no significant differences between non-exposed and exposed cohorts (Supplementary Table 2).

### Differences in FD predict clinical symptoms reported by individuals stationed in Havana

**Figure 2:**
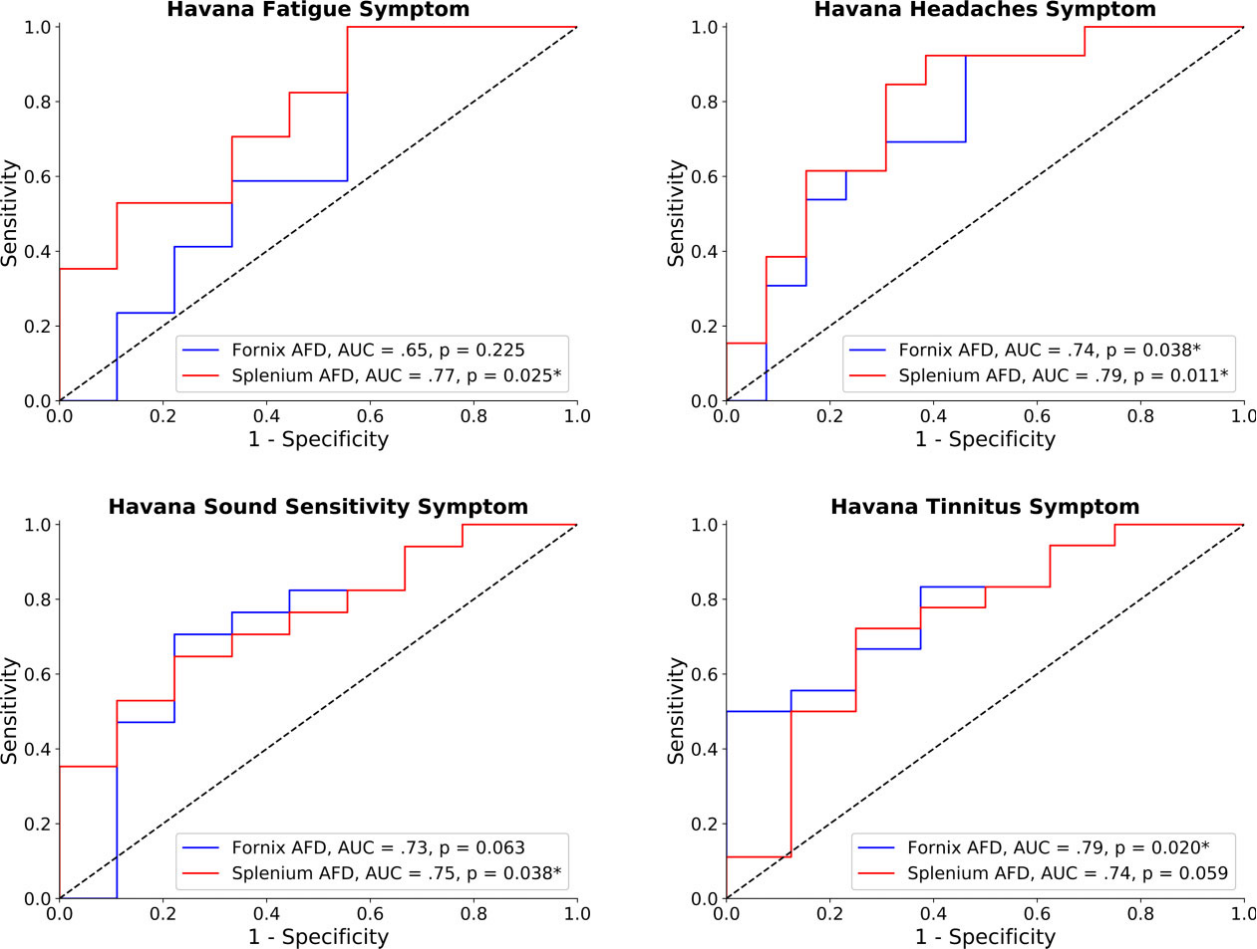
**Receiver operating characteristic analysis revealed that clinical symptoms are linked to FD reduction in the fornix and the splenium.** The reduction of FD in both structures was linked to the strength of migraine-like symptoms, whereas symptoms of fatigue and sound sensitivity were more strongly linked to the reduction of FD in the splenium only, and symptoms of Tinnitus to FD reductions in the fornix.

When testing the link between FD in the fornix and/or splenium and self-reported symptoms, FD was found to be predictive of several clinical symptoms, including headaches, fatigue, tinnitus and sound sensitivity (Table 1; Fig. 2). Notably, FD in both the fornix and the splenium was predictive of headaches—the most commonly reported symptom—with accuracy of 74 and 79%, respectively. Other frequently reported symptoms, such as tinnitus, sound sensitivity and fatigue could also be classified based on FD values in the fornix, splenium or both.

**Table 1:** Fibre density in the fornix and splenium and self-reported Havana symptoms

Symptom	No exposure (%)	Exposure (%)	Group differences^a^		ROC curve results^b^	
			Fornix	Splenium	Fornix	Splenium
Concentration	0 (0)	5 (31.25)	1.094, 0.286	1.073, 0.295	0.695, 0.182	0.743, 0.097
Memory	0 (0)	6 (37.5)	0.540, 0.595	0.603, 0.553	0.600, 0.465	0.650, 0.273
Blurred vision	1 (10)	7 (43.75)	1.083, 0.29	1.340, 0.194	0.681, 0.149	0.729, 0.067
Light sensitivity	0 (0)	5 (31.25)	1.115, 0.277	1.167, 0.256	0.667, 0.255	0.705, 0.162
Headaches	0 (0)	13 (81.25)	2.070, 0.050	2.63, 0.015	0.740, **0.038**	0.793, **0.011**
Tinnitus	0 (0)	8 (50)	2.256, 0.034	1.994, 0.059	0.792, **0.020**	0.736, 0.059
Sound sensitivity	0 (0)	9 (56.25)	1.885, 0.073	2.016, 0.056	0.725, 0.063	0.752, **0.038**
Vestibular	0 (0)	6 (37.5)	0.580, 0.568	0.729, 0.474	0.600, 0.465	0.642, 0.301
Sleep	1 (10)	9 (56.25)	1.490, 0.151	1.530, 0.14	0.681, 0.126	0.681, 0.126
Fatigue	0 (0)	9 (56.25)	1.476, 0.154	2.258, 0.034	0.647, 0.225	0.771, **0.025**

^a^ Data formatted as (*t*-value, *P*-value).

^b^ Data formatted as (area under curve, *P*-value).

Bold figures denote significances (*P* < 0.05)

### Connectivity alterations in white matter networks among exposed individuals

**Figure 3:**
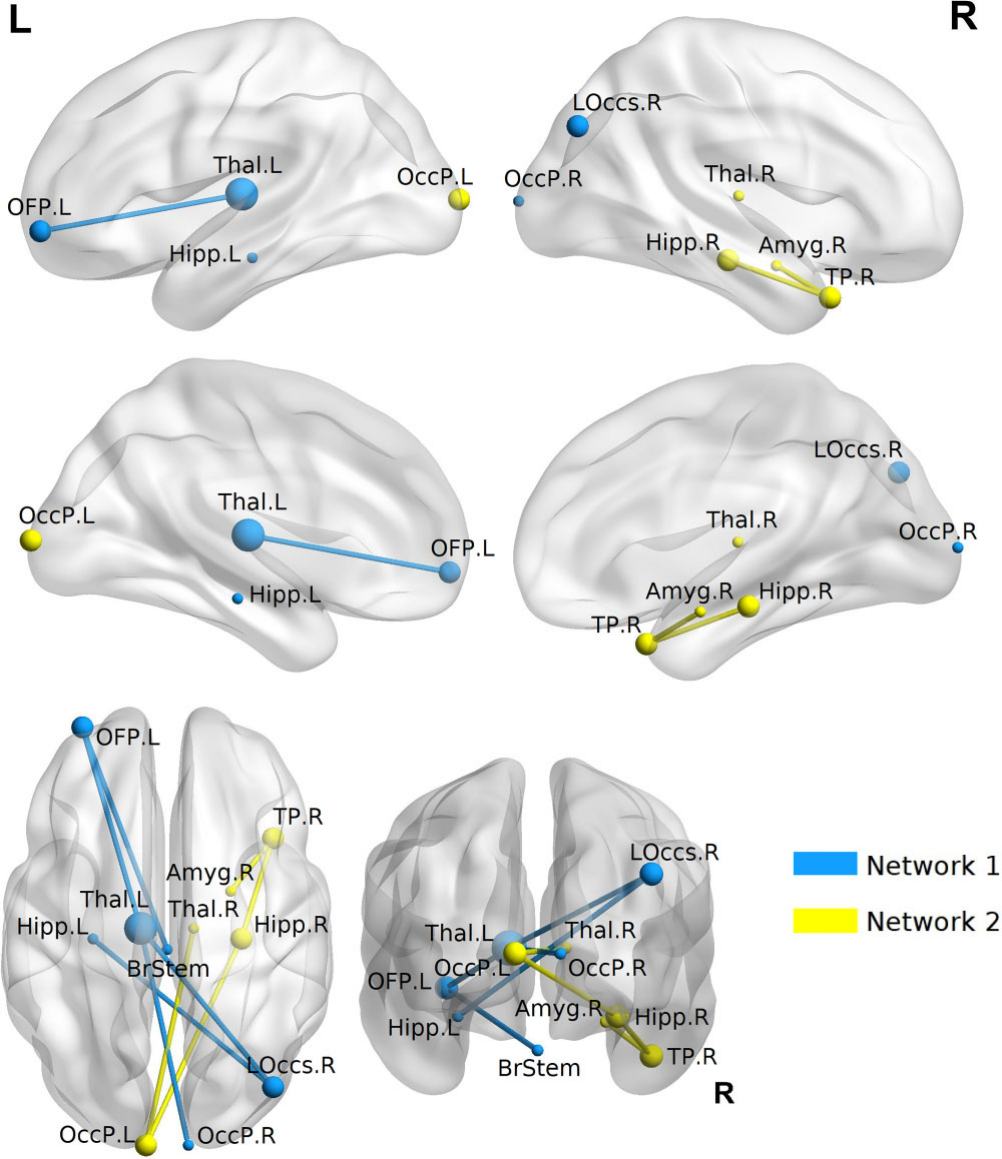
**Distinct network clusters were found to be significantly decreased in individuals stationed in Havana**. Group comparison using NBS revealed two networks of brain regions with significantly (FWE *P* < 0.05) decreased structural connectivity in exposed diplomats (*n* = 16), compared with unexposed individuals (*n* = 48). Brain node size indicates the degree of brain node within the significantly affected network. See Table 2 for full description of the affected edges, region names, *t*-values and network *P*-values.

The structural brain networks observed with probabilistic tractography demonstrated significantly reduced connectivity in individuals stationed at Havana. Whole brain group comparisons of structural connectomes carried out using NBS,^16^ revealed a significant (FWE *P* < 0.05) difference in two distinct networks. Both networks were comprised of connections within and between subcortical, sensory, orbitofrontal and temporal regions; and both networks had reduced connectivity between subcortical regions. All affected connections are summarized in Table 2 and Fig. 3.

**Table 2:** Structural white matter edges found to have significantly decreased SIFT2-weighted structural connectivity in the exposed cohort relative to the unexposed cohort

Node 1	Node 2	t-value
Network 1 (FWE *P* = 0.0174)		
Hippocampus left (Hipp_L)	Lateral occipital cortex, superior division right (LOccS_R)	3.16
Brain stem (BrStem)	Orbitofrontal pole left (OFP_L)	3.31
Lateral occipital cortex, superior division right (LOccS_R)	Thalamus left (Thal_L)	3.18
Occipital pole right (OccP_R)	Thalamus left (Thal_L)	3.4
Orbitofrontal pole left (OFP_L)	Thalamus left (Thal_L)	3.41
Network 2 (FWE *P* = 0.0298)		
Hippocampus right (Hipp_R)	Occipital pole left (OccP_L)	3.39
Occipital pole left (OccP_L)	Thalamus right (Thal_R)	3.26
Amygdala right (Amyg_R)	Temporal pole right (TP_R)	3.69
Hippocampus right (Hipp_R)	Temporal pole right (TP_R)	3.64

## Discussion

The findings presented in this work demonstrate that the Canadian diplomats and their families who were stationed in Havana show significantly lower WM integrity relative to a cohort of non-exposed diplomats and healthy controls. Exposed individuals were observed to have significantly decreased FD in the posterior fornix and in the splenium, and decreased network structural connectivity. Moreover, FD differences among diplomats correlated significantly with the duration of stay in Havana and were found to be correlated with self-reported clinical symptoms.

An analogous investigation conducted in the USA by Verma *et al.*^5^ has studied US Government personnel who reported neurological symptoms after serving in Havana. The study reported a profile of symptoms that was similar to our cohort, along with reductions in WM volume in frontal, occipital and parietal regions compared with controls. Using diffusion-weighted imaging coupled with DTI analysis, the study also showed significantly decreased MD in the inferior vermis of the cerebellum. No significant differences in whole-brain structural connectivity were reported. An important caveat is that in the American cohort, the studied individuals were recruited based on the presence of neurological symptoms and were tested long after leaving Havana. Other important limitations of the American study include the potential lower sensitivity of the DTI method in voxels with crossing fibres, and the lack of reporting of whole-brain statistical corrections.

The present study employed the FBA approach, which uses higher-order diffusion-weighted models to overcome the limitations of DTI analysis. This approach examines diffusion properties of individual fibre populations within a voxel instead of estimating a voxel-average measure.^6,35^ Moreover, FBA is sensitive to microstructural changes of WM tracts, including FD, FC or both FDC.^12^ Using this technique and a permutation method for correcting for multiple comparisons at a whole-brain level, we were able to avoid the ambiguities of previous investigations. The present study also recruited any Canadian individuals who were posted in Havana, without additional criteria based on the presence or absence of symptoms. Additionally, we were able to examine individuals shortly after return from Havana; defining the exposure group as individuals who have spent at least 30 days in Havana, and who underwent clinical and MRI testing within a month of their return to Canada.

Using the FBA approach, we show significant localized differences in WM properties (specifically FD) in both the fornix and the splenium. Moreover, FA analysis conducted using the whole-brain TBSS method revealed differences approaching significance in the fornix, splenium and the left superior corona radiata, partially corroborating our FBA findings. However, our cerebellar GM diffusion analyses did not replicate the findings by Verma *et al.*^5^ and no significant effects were observed in the cerebellar WM.

Decreased FD of WM structures, as observed in our study, is reflective of reduced density of axons within fibre tracts, potentially indicative of a reduction in the fibre’s capacity to relay information^12^ and axonal injury.^6^ While the relatively new FBA approach has yet to be studied in many pathologies, a study in TBI has demonstrated fibre density differences in WM structures such as the corpus callosum, cerebral peduncle and internal and external capsules.^6^ Notably, the splenium is the thickest and most posterior portion of the corpus callosum and has been reported to be the region most affected in TBI.^36,37^ Specifically, a DTI study by Rutgers *et al.*^38^ has demonstrated splenium injury (based on FA and MD abnormalities) following moderate and severe TBI. More recently, a DTI study by Ghodadra *et al.*^39^ has associated WM abnormalities in the midsplenium with post-traumatic headaches. Our study supports these findings, showing that reduced FD in the splenium is predictive of headaches and fatigue in individuals posted in Havana. The literature demonstrating the involvement of the splenium in headaches and TBI suggests that individuals posted in Havana appear to present TBI-like symptoms, which may correspond to a wide range of TBI severity.

The fornix is thought to play an important role in memory and cognition; and disruption of the fornix has been suggested to be a robust predictor for major memory deficits in neurodegenerative disorders, such as Alzheimer’s.^40,41^ However, in our study, disruption of the fornix was not associated with decreased memory performance but was associated with headaches and tinnitus. These findings are in agreement with reports linking the fornix to cortical spreading depression and the pathophysiology of migraine,^42,43^ through its role in serotonergic projections to the hippocampus.^43^ The link we observed between the fornix and tinnitus is indirectly supported by neuroimaging studies associating tinnitus generation/suppression with activity in auditory and non-auditory brain regions, including limbic brain structures such as the hippocampus and amygdala.^44,45^ Further multimodal studies are needed to better ascertain the potential clinical relevance and implications of the abnormalities observed in the splenium and the fornix in our cohort.

Probabilistic tractography revealed that exposed individuals had significantly decreased structural connectivity in two networks of subcortical–cortical connections, consisting of regions involved in visual processing, memory, learning and emotion processing. Tractography analysis also partially corroborated our FBA findings of splenium abnormalities, as some of the edges with lower structural connectivity involve passage through the splenium (e.g. the hippocampal and thalamic connections to the occipital and parietal cortex).^46^ While the clinical implications of decreased structural connectivity should be subject of more investigation, it serves as further potential evidence of WM abnormalities that resemble diffuse axonal injury in TBI studies.^15^

The diverse clinical assessments carried out in our study proved to be beneficial in capturing the wide array of symptoms in our participants. Our study conducted visual and audio-vestibular assessments, a thorough assessment of medical history, and a wide range of symptom questionnaires. We were, thus, able to test the juxtaposition between reported symptoms and neuroimaging findings, something that has not been tested by any of the studies on this topic so far. We stress the importance of testing the overlap between self-reported symptoms and neuroimaging results, since self-reported symptoms are usually the main information guiding the care of affected individuals by their physicians.

The source of the shared symptoms affecting both American^1,2,5^ and Canadian^4^ diplomats, as well as tourists,^47^ is still to be deciphered. Some have theorized that acoustic attacks were behind the illness—a theory that has captured considerable media attention^2,3^—but the evidence remains inconclusive. In previous work carried out by our team,^4^ a hypothesis of exposure to neurotoxins was introduced as the potential cause for the reported symptoms. Findings of reduced cholinesterase enzyme activity and presence of cholinesterase-inhibiting insecticides pointed to low-dose exposure to organophosphates as the potential source of acquired brain injury.^4^ These findings coincided with documented evidence of extensive fumigations with organophosphorus and other insecticides in Cuba to fight the spread of Zika virus from 2016 to 2018.^4^ In this context, the loss of fornix fibre density reported here is consistent with a cholinergic toxicity, as it contains cholinergic fibres originating in the basal forebrain projecting to the hippocampal formation. The present findings corroborate the possibility that damage may have occurred, but a longitudinal prospective study will be necessary both for validation and to identify the cause. This study was conducted in the same cohort of Canadians, who spent over a month in Cuba between 2016 and 2018. We further show that more severe WM disruption was correlated with longer stays in Havana, suggesting that prolonged exposure may have a cumulative. Our neuroimaging results reveal WM differences that are correlated to reported symptoms. Further investigations should be carried out to confirm and delineate the cause and mechanism, and to test the mechanisms potentially linking exposure to organophosphates and WM alterations or other potential causes.

Limitations of this study include the relatively small sample size of exposed individuals. The reported symptoms by Canadians posted in Havana have led to a drastic reduction in the number of individuals being posted in Havana in the past 2 years. To address this limitation, diplomats from the non-exposed cohort were grouped with a larger cohort of healthy controls when performing statistical comparisons with exposed individuals. Findings were subsequently tested in a *post hoc* analysis within the exposed group, to confirm the results. Given that this is a unique sample, a multimodal and quantitative assessment of the neurological manifestations with convergent findings may shed further light on the underlying mechanisms of these pathologies. An important component that needs further study is whether individuals will be affected by long-term cognitive impairment and whether neuroimaging assessments can attribute cognitive symptoms to differences in brain structure or brain network connectivity. The recruitment and randomization process for this study was dictated by the specific nature of the symptoms and hence imposes a possible limitation. Nevertheless, the whole brain corrected significant effects and the association between neurological effects, symptoms and duration of exposure indicate that the effects are specific to exposure and not obfuscated by the limitations of study randomization.

Overall, our results reveal region-specific decline in white matter FD and network structural connectivity in Canadian individuals who spent over a month in Havana between 2016 and 2018. Using advanced diffusion-weighted imaging techniques, we identified microstructural alterations in the fornix, which corresponded to headaches, and tinnitus and microstructural alterations in the splenium, that corresponded to headaches and fatigue. We also found evidence of disrupted structural network connectivity in commissural thalamic and hippocampal projections to posterior regions of the brain. The specific cause of the observed WM differences remains unknown.

## Supplementary Material

fcac053_Supplementary_Data

## Abbreviations

ACT =: anatomically constrained tractography
CSD =: constrained spherical deconvolution
dMRI =: diffusion-weighted MRI
DTI =: diffusion tensor imaging
FA =: fractional anisotropy
FBA =: fixel-based analysis
FC =: fibre cross-section
FD =: fibre density
FDC =: fibre density and cross-section
FOD =: fibre orientation distribution
FOV =: field of view
FWE =: family-wise error correction
GM =: grey matter
MD =: mean diffusivity
NBS =: network-based statistics
ROC =: receiver operating characteristic
ROI =: region of interest
SIFT2 =: spherical-deconvolution filtering of tractograms (revised)
SS-EPI =: single-shot echo-planar imaging
TBI =: traumatic brain injury
TBSS =: tract-based spatial statistics
TE =: MRI echo time
TR =: MRI repetition time
WM =: white matter
