# Supplementary material for: Symptoms reported by Canadians posted in Havana are linked with reduced white matter fibre density
Source: Brain Commun. 2022 Mar 7;4(2):fcac053. doi: 10.1093/braincomms/fcac053 (PMC9050567; doi:10.1093/braincomms/fcac053)
Supplement: fcac053_Supplementary_Data [file fcac053_supplementary_data.docx]

## Supplementary materials

**Supplementary Table 1.** Study information on duration of exposure and time between exposure and assessment. Note that the total N=26, three individuals were tested before and after exposure.

|  |  | **No**  **Exposure**  N=10 | **Recent**  **Exposure^1^**  N=6 | **Remote Exposure^2^**  N=11 |
| --- | --- | --- | --- | --- |
|  | Male (%) | 4 (40) | 3 (50) | 4 (36) |
|  | Female (%) | 6 (60) | 3 (50) | 7 (64) |
| Age (Years) | Median (Range) | 48.5 (28-64) | 36 (29-55) | 41 (25-61) |
| Duration of Exposure (Weeks) | Median  (Range) | N/A | 20.64  (15.29-56.57) | 152.14  (5.43-198.00) |
| Time Between Exposure and Assessment (Weeks) | Median  (Range) | N/A | 0.50  (0.14-2.29) | 56.57  (8.57-82.57) |

^1^Assessed within one month of their return from Havana, Cuba.

^2^Assessed more than one month after their return from Havana, Cuba

**Supplementary analysis on cerebellar structural connectivity**

Following the diffusion-weighted MRI (dMRI) preprocessing as described in ‘MRI image preprocessing’ in the ‘Methods’ section of the main paper, all diffusion images underwent further processing tailored toward statistical comparisons using fractional anisotropy (FA) and mean diffusivity (MD). These FA and MD images were created by fitting a tensor model and then extracting the brains. Using FSL’s FNIRT^1^, registration warps were computed to align and subsequently warp FA images to template space. The MD images were also warped to template space using the computed warp matrices. Warped FA images were used to compute a skeleton in template space and voxel-wise statistical analysis of the FA data was carried out using tract-based spatial statistics (TBSS)^2^. Cerebellar ROIs were defined using Yeo’s seven-network parcellation^3,4^ and used to compute mean MD values in functionally defined grey matter regions of the cerebellum for all participants. This analysis is analogous to that of Verma *et al.*^5^, thus only six networks in the cerebellum were assessed, resulting in a total of 12 bilateral ROIs.

## Supplementary results

**Supplementary Figure 1 TBSS results showed tracts approaching significance that are in line with our main findings.** Whole-brain statistical comparisons using TBSS and FSL’s randomise script revealed three clusters of voxels (shown in blue-red gradient) where the exposed cohort had decreased FA than the unexposed cohort, with a significance of 0.16 < FWE *p* < 0.30. The three clusters of voxels were found in the fornix, the splenium of the corpus callosum, and the left superior corona radiata.


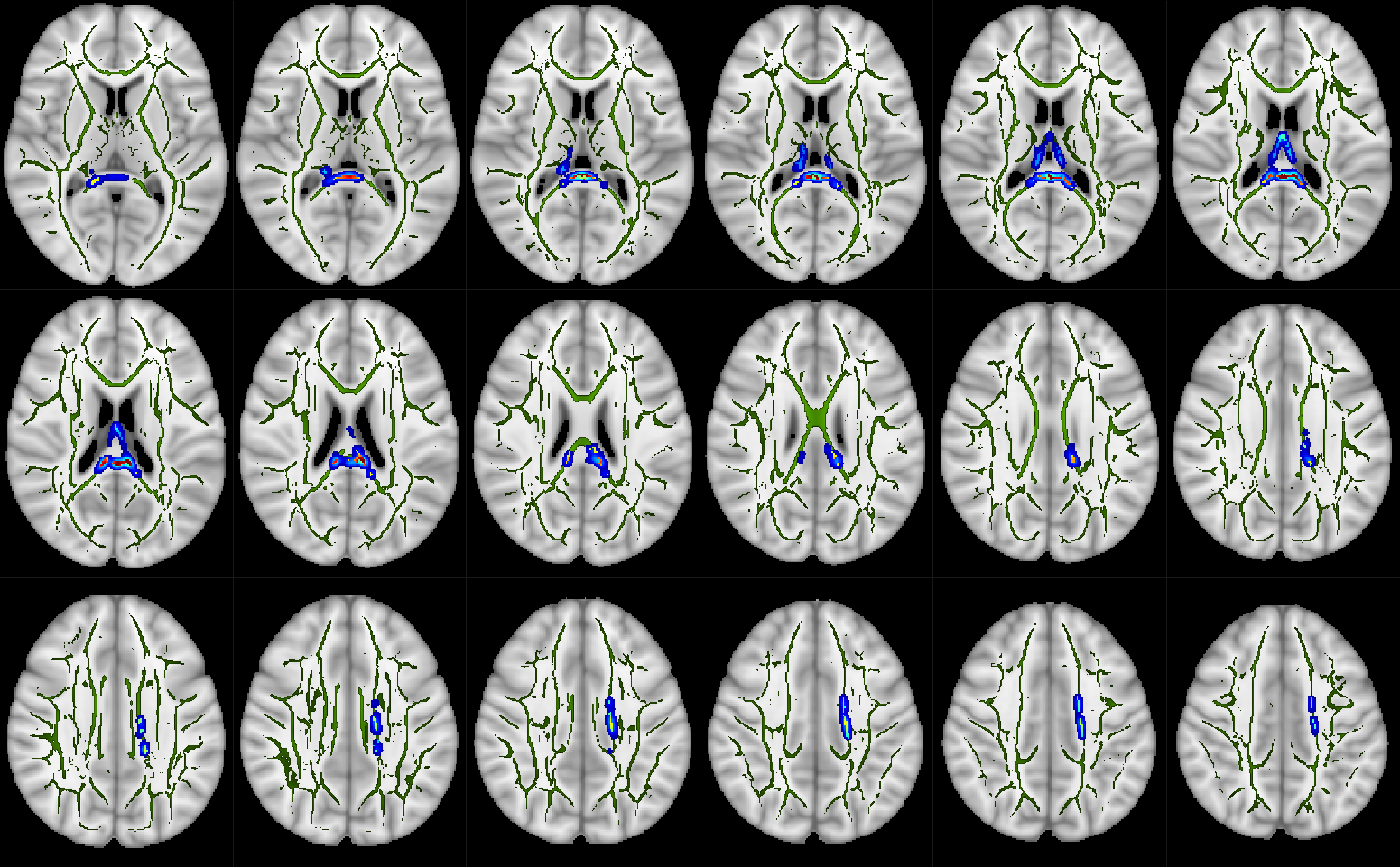


**Supplementary Table 2 Functionally defined grey matter regions in the cerebellum did not show significant differences in mean diffusivity**

| **Functional Network** | **t-value** | ***p*-value** |
| --- | --- | --- |
| Somatomotor Network, Left Cerebellum | -0.834 | 0.408 |
| Somatomotor Network, Right Cerebellum | -0.790 | 0.433 |
| Dorsal Attention Network, Left Cerebellum | -0.454 | 0.652 |
| Dorsal Attention Network, Right Cerebellum | -0.880 | 0.382 |
| Ventral Attention Network, Left Cerebellum | -0.526 | 0.601 |
| Ventral Attention Network, Right Cerebellum | -0.850 | 0.399 |
| Limbic Network, Left Cerebellum | -1.189 | 0.239 |
| Limbic Network, Right Cerebellum | -1.204 | 0.233 |
| Frontoparietal Network, Left Cerebellum | -0.255 | 0.799 |
| Frontoparietal Network, Right Cerebellum | -0.133 | 0.894 |
| Default Network, Left Cerebellum | -0.387 | 0.700 |
| Default Network, Right Cerebellum | -0.321 | 0.749 |

# References

1. Jenkinson M, Beckmann CF, Behrens TE, Woolrich MW, Smith SM. Fsl. *Neuroimage*. Aug 15 2012;62(2):782-90. doi:10.1016/j.neuroimage.2011.09.015

2. Smith SM, Jenkinson M, Johansen-Berg H, et al. Tract-based spatial statistics: voxelwise analysis of multi-subject diffusion data. *Neuroimage*. Jul 15 2006;31(4):1487-505. doi:10.1016/j.neuroimage.2006.02.024

3. Buckner RL, Krienen FM, Castellanos A, Diaz JC, Yeo BT. The organization of the human cerebellum estimated by intrinsic functional connectivity. *J Neurophysiol*. Nov 2011;106(5):2322-45. doi:10.1152/jn.00339.2011

4. Yeo BT, Krienen FM, Sepulcre J, et al. The organization of the human cerebral cortex estimated by intrinsic functional connectivity. *J Neurophysiol*. Sep 2011;106(3):1125-65. doi:10.1152/jn.00338.2011

5. Verma R, Swanson RL, Parker D, et al. Neuroimaging Findings in US Government Personnel With Possible Exposure to Directional Phenomena in Havana, Cuba. *JAMA*. Jul 23 2019;322(4):336-347. doi:10.1001/jama.2019.9269
